# MetaTrans: an open-source pipeline for metatranscriptomics

**DOI:** 10.1038/srep26447

**Published:** 2016-05-23

**Authors:** Xavier Martinez, Marta Pozuelo, Victoria Pascal, David Campos, Ivo Gut, Marta Gut, Fernando Azpiroz, Francisco Guarner, Chaysavanh Manichanh

**Affiliations:** 1 Digestive Research Unit, Vall d’Hebron Research Institute, Barcelona, 08035, Spain; 2 CNAG-CRG, Centre for Genomic Regulation (CRG), Barcelona, 08028, Spain; 3 Universitat Pompeu Fabra (UPF), 08002, Barcelona, Spain; 4 CIBERehd, Instituto de Salud Carlos III, Madrid, 28029, Spain

## Abstract

To date, meta-omic approaches use high-throughput sequencing technologies, which produce a huge amount of data, thus challenging modern computers. Here we present MetaTrans, an efficient open-source pipeline to analyze the structure and functions of active microbial communities using the power of multi-threading computers. The pipeline is designed to perform two types of RNA-Seq analyses: taxonomic and gene expression. It performs quality-control assessment, rRNA removal, maps reads against functional databases and also handles differential gene expression analysis. Its efficacy was validated by analyzing data from synthetic mock communities, data from a previous study and data generated from twelve human fecal samples. Compared to an existing web application server, MetaTrans shows more efficiency in terms of runtime (around 2 hours per million of transcripts) and presents adapted tools to compare gene expression levels. It has been tested with a human gut microbiome database but also proposes an option to use a general database in order to analyze other ecosystems. For the installation and use of the pipeline, we provide a detailed guide at the following website (www.metatrans.org).

In the last decade, next-generation sequencing technologies have allowed sequencing at a very low-cost and have thus boosted the use of meta-omic approaches to study microbial communities. To date, the main challenge is to develop, create, and optimize reliable tools that take advantage of current multi-threading computers to analyze the huge amount of data generated by high-throughput sequencing technologies.

Over the last decade, the human microbiome has been the focus of important international consortia such as the Human Microbiome Project, a NIH initiative, and MetaHIT, a European consortium. These consortia have deposited catalogues of microbial genes in an unprecedented amount^1^,^2^.

Metagenomics aims at cataloging the genes present in a sample, while the study of RNAs, called metatranscriptomics, provides an opportunity to gain insights into the functionality of microbial communities. By assessing the genes expressed by the microbial community, metatranscriptomics gives a mechanistic understanding of inter-community relationships and the crosstalk between a microbial community and its host^3^,^4^. Previous transcriptomic^5^ and metatranscriptomic^6^,^7^,^8^ studies developed various approaches to analyze RNA-Seq experiments; however, the particularity of distinct experimental methods hinders the development of a generic pipeline that covers all possible scenarios. It is important that such tools be not only flexible and adaptable but also efficient, both in terms of runtime and memory footprint.

Here we present a downloadable, open-source, effective and efficient metatranscriptomic pipeline developed for a paired-end RNA-Seq analysis and easily adaptable to other high-throughput experiments. Given the rapid emergence of research into metatranscriptomics, additional bioinformatics tools are likely to be developed for specific tasks in the near future and will probably serve to improve our pipeline. Thus, we designed the pipeline in order to facilitate the inclusion of such third-party tools in each of its stages.

Our pipeline was designed to perform two types of RNA-Seq analyses, namely those addressing 16S rRNA taxonomy and gene expression. To test the present metatranscriptomic pipeline, we analyzed synthetic mock communities, twelve fecal samples collected from eight individuals obtained from a previous study^9^ and from an unpublished one. For four individuals, stool samples were collected and intestinal volume of gas was measured before and after a flatulogenic diet challenge of three days. In the present study, we believe that combining 16S rDNA, 16S rRNA and mRNA data can provide a new perspective of the factors involved in the origin of flatulence. Using these samples, we extracted total RNA, performed an rRNA removal step in a set of four samples and prepared cDNA libraries for paired-ends sequencing (to increase the read fragment and improve the read mapping) by Illumina machines (http://arxiv.org/pdf/1303.3997v2.pdf), which produced an average of 22 million paired-reads of short-length (76 bp) per sample. The reads were mapped against functional databases. We also compared two methods of taxonomic analysis; one using 16S rDNA V4 amplicons and the other 16S rRNA extracted from total RNA.

## Results

### Validation of our pipeline

**Figure 1:**
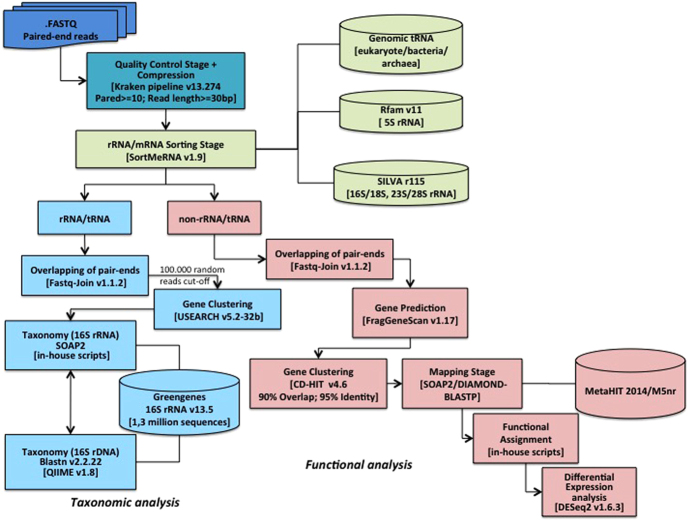
Flow diagram of the metatranscriptomic pipeline. The raw paired-end reads were subjected to quality control and adjustment using the FastQC tool and Kraken pipeline (turquoise boxes). The rRNA/tRNA reads were then separated from the non-rRNA/tRNA reads using SortMeRNA software (green boxes), for taxonomic (clear blue boxes) and functional analyses (pink boxes), respectively. For the taxonomic analysis, the reads were mapped against the 16S rRNA Greengenes v13.5 database using SOAP2. For the functional analysis, the reads were subjected to the FragGeneScan to predict putative genes before being mapped against a functional database (MetaHIT-2014 or M5nr) also using the SOAP2 tool (see methods for details).

As described in Fig. 1, our pipeline, consisting of four major steps (filtering, sorting, and functional and taxonomic analyses), included tools implemented with multi-threading options and used the most updated functional human gut database (MetaHIT-2014)^2^.

**Figure 2:**
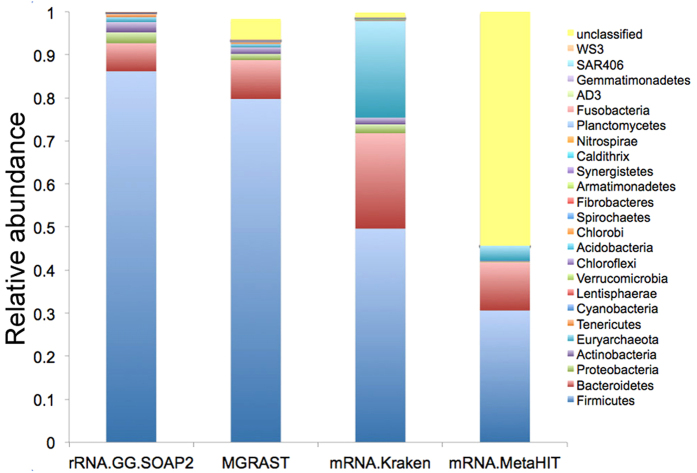
Comparison of taxonomic classification methods. Taxonomic assignment in terms of abundance for the fecal sample #1_BF using 16S rRNA sequences mapped with SOAP2 against Greengenes (rRNA.GG.SOAP2), the whole sample analyzed with MG-RAST (MG-RAST), mRNA assigned with Kraken (mRNA.Kraken) and mRNA mapped with SOAP2 against MetaHIT-2014 (mRNA.MetaHIT). This bar plot shows similar taxonomic profiles between rRNA.GG.SOAP2 and MG-RAST whereas they differ with mRNA.MetaHIT and mRNA.Kraken. Unclassified reads are more abundant in the last method.

In order to validate MetaTrans in terms of taxonomic analysis, we compared different available methods such as 1) 16S rRNA sequences analyzed with the SOAP2 tool^10^ and the Greengenes database^11^; 2) total RNA analyzed with MG-RAST^12^; 3) mRNA sequences analyzed with the Kraken tool^13^; 4) mRNA analyzed with SOAP2 and the MetaHIT-2014 database. Kraken is a taxonomic sequence classifier that assigns taxonomic labels to short DNA reads. To classify a sequence, Kraken maps each k-mer in the sequence to the lowest common ancestor (LCA) of the genomes that contain that k-mer in a database (NCBI-bacterial/archaeal genomes). For rRNA identification, MG-RAST uses a BLAT similarity search for the longest cluster representative against the M5rna database that includes SILVA, Greengenes and RDP databases. To compare the different methods, we used all the sequences of one of our processed fecal sample (#1_BF), for which, we generated 39 millions paired-end reads. After processing the reads by MetaTrans, we recovered 1.6 millions of 16S rRNA paired-end sequences and 700000 mRNA hit against the MetaHIT-2014 database that were then used for the methods comparison. As shown in the Fig. 2, 16S rRNA sequences analyzed with the SOAP2 tool and the Greengenes database (rRNA.GG.SOAP2) presented at the phylum level very similar results with those of the MG-RAST server and displayed very low proportion of unclassified reads (<5%). mRNA analyzed with Kraken and the NCBI bacterial database showed also low proportion of unclassified reads (1%) but higher relative abundance of Euryarchaeota than the two previous methods, which could be due to the higher copy number of 16S rRNA gene found in Bacteria compared to Archeae^14^. Only mRNA analyzed with SOAP2 and the MetaHIT-2014 database (mRNA.MetaHIT) presented a very high percentage of unclassified reads.

**Figure 3:**
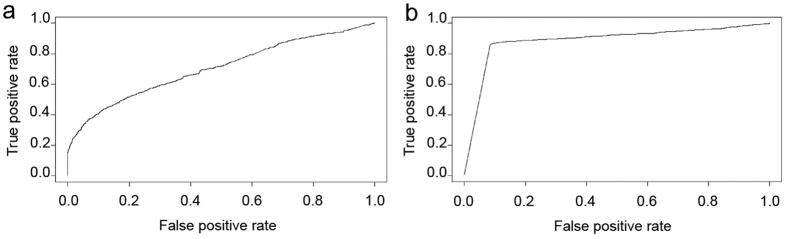
Performance of MetaTrans for analyses of mock community simulations ROC curves of (**a**) taxonomic and (**b**) functional mock community simulations, with 50 and 100 samples, respectively. AUC of 0.704 and of 0.887 were obtained for taxonomic and functional simulations, respectively.

To assess the accuracy of MetaTrans for taxonomic profiling, we also constructed two synthetic mock communities of 25 samples each. We applied a differential expression such that 20% of the genes presented a 4-fold overexpression and 20% a 4-fold underexpression between the two communities and the sensitivity and specificity of MetaTrans were evaluated using a receiver operating characteristic (ROC) curve (see Methods section; Fig. 3a). We obtained an AUC (area under the curve) of 0.704, which showed a fair accuracy of the method.

In order to validate MetaTrans in terms of functional analysis between two microbial communities, we also constructed two mock communities of 50 samples each and simulated a differential gene expression between the two communities as described above. Each sample contained 1000 genes randomly selected from five microorganisms commonly found in the gut microbiome (see in Methods section). As for the simulation of 16S rRNA dataset, we evaluated the performance of MetaTrans using a ROC curve (Fig. 3b). We obtained an AUC of 0.887, which showed a good accuracy of the method.

To test our pipeline in terms of functional analysis with real metatranscriptomic data, we recovered and processed part of the dataset published in a recent study^6^. This dataset consisted of paired-end reads obtained from the content of a human small intestine sample (42.2 million sequence reads for both ends). For these analyses, our pipeline was adapted to match the reads to the COG database (Clusters of Orthologous Groups, containing about 190,000 annotated functions and 25 categories of functions) using BLASTP, as performed in Leimena *et al*. We obtained all the 23 functional categories as described in Leimena *et al*. and in similar proportions (Supplementary Fig. S1).

### Experimental design

To test our pipeline with RNA-seq newly generated, we performed RNA sequencing in two types of experimental designs: “total RNA” and “rRNA removal” experiments. The objective of the “total RNA sequencing” experiment was to recover both the functional and taxonomic profile of each active microbial community in an unbiased manner. We performed this experiment on eight stool samples from four individuals that were collected in a previous study^9^. Moreover, in order to detect functional variations for each participant, samples were collected before and immediately after three days of a flatulogenic diet, as detailed in the Methods section. We then compared the 16S rRNA sequences with the 16S rDNA sequences that we recovered from our previous study^9^ after PCR amplification of extracted genomic DNA of the same samples. We envisaged that this comparison would indicate whether the microbes detected by the 16S rDNA gene survey were also those functionally active. The objective of the “rRNA removal” experiment was to test how the rRNA depletion step would increase the recovery of number of expressed genes. This experiment was performed on four additional stool samples obtained from four individuals.

As paired-end reads have been shown to recover fewer false positives than single ones^15^, we assembled, when possible, the single end reads using the Fastq-Join program before performing gene prediction by FragGenScan (Fig. 1).

### Data and output descriptions

The two experiments generated the following datasets: 318 million paired-end reads (76 bp) generated from the “total RNA” experiment (about 20 million paired-reads per sample; Supplementary Table S1) and 219 million paired-end reads (76 bp) generated from the “rRNA removal” experiment (about 27 million paired-reads per sample; Supplementary Table S2). For the “total RNA” experiment, we recovered an average of 78% high quality reads, 74% of rRNA/tRNA and 4.3% of non-rRNA/tRNA (e.g. potential mRNA), as expected. For the “rRNA removal” experiment, we obtained 55% of high quality reads, 2.7% of rRNA/tRNA and 52.3% of potential mRNA. As expected the proportion of potential mRNA recovered from “rRNA removal” experiment was 10 fold higher than in the “total RNA” experiment. However, the median number of unique orthologous IDs was only 1.27 fold only higher in the “mRNA removal” experiment (11541 versus 9032). Furthermore, we observed that the overlapping step allowed recovery of a longer read length for 42% of the non-rRNA/tRNA reads for the two experiments.

### Are your computer features a bottleneck?

Metatranscriptomic as well as metagenomic approaches are computationally very expensive (CPUs and RAM). In order to speed up the analysis, our pipeline was optimized by means of multi-threading software. In order to optimize the runtime, we tested several aligner tools such as DIAMOND-BLASTP^16^, SOAP2^10^ and BLASTP^17^ to map one of our dataset (#1_BF) against the MetaHIT-2014 database, containing human gut microbiome genes. The three tools provided very similar number of matched eggNOG IDs (Supplementary Fig. S2). However, SOAP2 and DIAMOND-BLASTP were 6600 and 480 fold much faster than BLASTP, respectively. We finally implemented SOAP2 and DIAMOND-BLASTP in our pipeline. The bottleneck still remains in the first steps of the analysis, in particular for the sorting and clustering steps. Therefore, to be able to perform these analyses in a reasonable timeframe, we recommend a minimum of 10 CPUs and 16 GB of RAM (size of the database or the query to be loaded). As an example, to analyze a sample, for which 39 millions paired-end reads were generated and about 1 million of potential genes were sorted out, 2 hours and 21 min was required with the following settings: 10 CPUs and 16 GB of RAM. The cost of a current computer with these features could approximate 3000 dollars.

### Taxonomic analysis of our dataset

**Figure 4:**
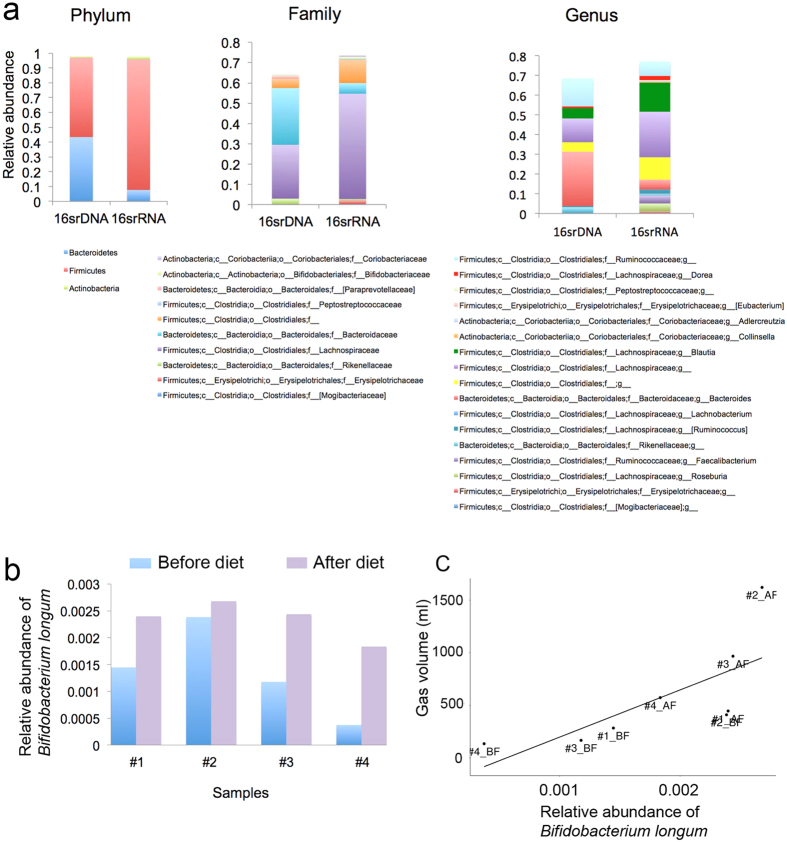
Taxonomic analysis at the DNA and RNA levels. (**a**) Significant differences between relative mean abundance of the 16S rRNA and 16S rDNA libraries at the phylum, family and genus levels (q-value < 0.05). (**b**) Effect of diet at the RNA level on the increase in relative abundance of *Bifidobacterium longum* (P < 0.05). (**c**) Correlation between volume of gas and relative abundance of *Bifidobacterium longum* (r = 0.92; P = 0.002; Spearman).

To describe the active microbial composition of our stool samples from the “total RNA” experiment, we mapped the reads labeled as rRNA/tRNA against the Greengenes (v13.5) 16S rRNA database. To speed up the taxonomic analysis, we randomly selected a reasonably high number of reads, namely 100000, a much higher number than most studies performing 16S rRNA gene surveys. At the phylum, family, genus and species levels, we identified 7, 29, 49 and 70 groups of microbes, respectively, with at least 1% of sequences in at least one sample, in order to avoid false positives (Supplementary Fig. S3). Four phyla accounted for 99.3% of the dataset: Firmicutes (87%), Bacteroidetes (8.1%), Actinobacteria (1.9%), and Proteobacteria (1.8%). At the family level, Lachnospiraceae (52.2%), Ruminococcaceae (18%), unknown Clostridiales (11%), Bacteroidaceae (5%), Erysipelotrichaceae (1.9%), Clostridiaceae (1.9%), and Porphyromonadaceae (1.1%) accounted for 91% of the total relative abundance. Comparative 16S rDNA and 16S rRNA sequence analysis indicated significant differences (q-value < 0.05, Kruskal-Wallis) between relative mean abundance of the 16S genes detected at all phylogenetic levels from phylum to species, suggesting that the 16S rDNA survey did not provide the profile of the active microbial community. Indeed, at RNA level, Firmicutes might be a more dominant part of the metabolically active bacteria than suggested at DNA level (average of 87% in rRNA vs. 53% in rDNA sequence libraries) (Fig. 4a). At the family level, Lachnospiraceae (52% for rRNA vs. 26% for rDNA) was a significantly more active component than Bacteroidaceae (5.2% for rRNA vs. 28% for rDNA). At the genus level, an unknown Lachnospiraceae, *Blautia* (a Lachnospiraceae genus) and an unknown Clostridiales predominated the rRNA libraries, with a total mean of 50%. In contrast, in the rDNA libraries, *Bacteroides,* an unknown Ruminococcaceae and an unknown Lachnospiraceae totaled 50%. Interestingly, most 16S rDNA surveys and metagenomic approaches previously proposed Bacteroidaceae as a major actor in gut function and revealed Lachnospiraceae as the most active group of microbes (Human Microbiome Project). Indeed, members of the Lachnospiraceae family have been linked to obesity and protection against colon cancer in humans. This protective function is mainly due to the association of many species within the group with the production of butyric acid that is important for both microbial and host epithelial cell growth^18^.

To compare the number of taxa present at DNA and RNA levels, we first normalized the number of sequence reads per library to 1,952 and used the Friedman test. We observed that the flatulogenic diet caused an increase in Bifidobacteriaceae and more specifically *Bifidobacterium longum* (P < 0.05), at the RNA level (Fig. 4b) but not at the DNA level. As Bifidobacteriaceae is well-known as a saccharolytic bacterial group, this result would be consistent with a consumption of a flatulogenic diet.

### Functional analysis of our dataset

To characterize the active microbial functions of the eight stool samples from the “total RNA” experiment, reads labeled as non-rRNA/tRNA were subjected to FragGeneScan to predict putative genes and were then mapped against a known protein database, namely MetaHIT-2014 (Integrated Gene Catalog from human gut microbiome)^2^. The MetaHIT-2014 database was annotated following the Kyoto Encyclopedia of Genes and Genomes (KEGG) and the evolutionary genealogy of genes non-supervised orthologous group (eggNOG) databases and it contains 9.9 million non-redundant genes identified in the human gut. Mapping against MetaHIT-2014 allowed us to assign an average of 1.85% (731,527 of reads on average) of the high quality reads to an average of 206,330 non-redundant MetaHIT IDs or genes (ranging from 85,343 to 288,398) and 25 clusters of orthologous groups (COGs) or functional categories per subject. Supplementary Table S3 shows the distribution of the annotated orthologous groups, with carbohydrate transport and metabolism being the most abundant known functional group, as expected for the human gut microbiome^2^.

**Figure 5:**
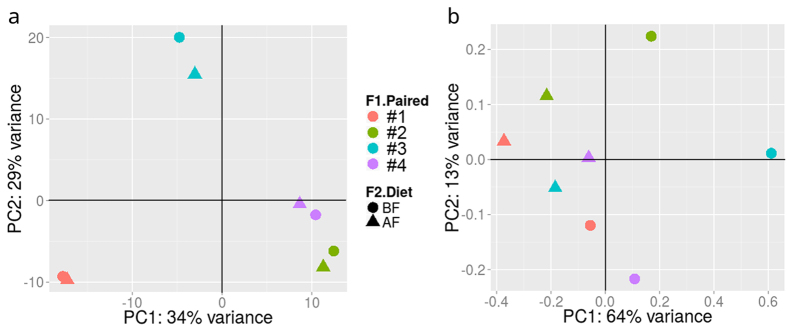
Principal Component analysis of the matrix of eggNOG IDs (**a**) or COG functional categories (**b**) showed that the two samples, before and after diet, clustered when all functions were taken into account but not when categories of functions were considered.

Further analyses were performed using the DESeq2 package. To detect differentially expressed functions and categories of functions, we applied a Principal Component Analysis to the matrix of abundance count generated after the mapping step. In terms of global eggNOG IDs, the two samples from each subject clustered and were located far from those of the other subjects (PC1 = 34%; Fig. 5a), while in terms of functional categories, samples clustered according to the effect of the flatulogenic diet for three of the subjects (PC1 = 64%; Fig. 5b). These results suggested that each individual has a specific set of functions and that a flatulogenic diet influenced families of functions.

**Figure 6:**
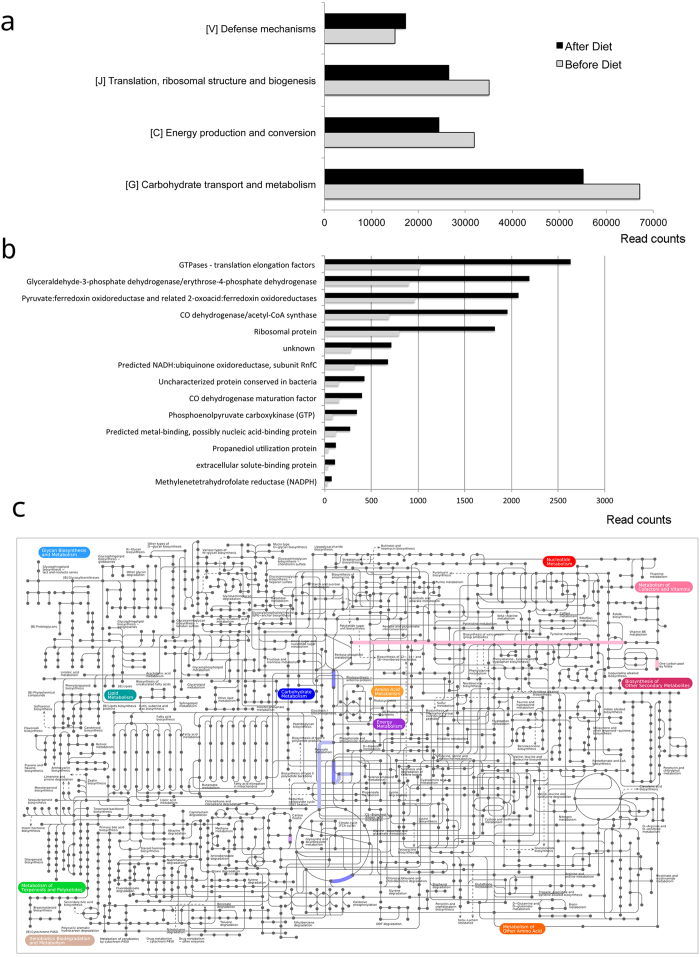
Effect of a flatulogenic diet on gene expression. Functional categories (**a**) that were up and down-regulated and Orthologous IDs (**b**) that were down-regulated as an effect of the diet challenge (q-value < 0.05). (**c**) The down-regulated functions plotted into a metabolic pathway network using the iPath2 tool.

To identify differentially regulated functions or categories of functions, we computed “FoldChange” on a matrix of raw count functions before and after the flatulogenic diet and then tested whether the mean of the log ratios was significantly different from zero following false-discovery rate (FDR) correction (indicating a pattern of up- or down-regulation of functions). As an effect of diet, we observed a significant increase in one category (q-value < 0.01) involved in “Defense mechanisms” and three down-regulated categories involved in “Translation, ribosomal structure and biogenesis”, “Energy production and conversion” and “Carbohydrate transport and metabolism” (Fig. 6a). We also identified 27 down-regulated orthologous IDs (with a log2FoldChange <−1; q-value < 0.01) (Fig. 6b). These were plotted into a network of metabolic pathways using the iPath2 tool (Fig. 6c). Among the up-regulated functions, the most abundant was found to be involved in bacterial secretion (Type IV secretory pathway, VirD4 components). The most abundant down-regulated functions were involved in translation (ribosomal protein and GTPases - translation elongation factors), glycolysis (GAPDH - Glyceraldehyde-3-phosphate dehydrogenase), nucleotide metabolism, vitamin B6 biosynthesis, energy metabolism, and CO dehydrogenase/acetyl-CoA synthase. The latter is central to the acetate production pathway.

### Correlation with gas production

To assess the link between a flatulogenic diet and intestinal gas production, we correlated the microbiome composition and functions with the volume of gas produced by the subjects and measured before and after the flatulogenic diet. The volume of intestinal gas, was found, at the DNA level, significantly and positively correlated with *Blautia* (r = 0.83; P = 0.01), a genus belonging to the Firmicutes phylum. Interestingly, several species belonging to this genus such as *Blautia hydrogenotrophica*, are capable of metabolizing H2/CO2 to acetate^19^. At the RNA level, only *Bifidobacterium longum* was positively correlated with the volume of gas (r = 0.92; P = 0.002; Fig. 4c). At the level of categories of functions, we observed that the volume of intestinal gas was significantly and positively correlated with two functional categories: “Inorganic ion transport and metabolism” and “Extracellular structures”; and negatively correlated with one functional category: “Cell motility”. Ninety-one orthologous IDs such as those involved in amino acids metabolism presented a significant positive correlation with the volume of gas, meanwhile 14 orthologous IDs such as those involved in energy metabolism were negatively correlated (Supplementary Table S4).

### Comparison with an existing web application server

In order to compare the results of our pipeline with those of MG-RAST, one of the few web application servers for metagenomic and metatranscriptomic analysis, we loaded one of our dataset (#1_BF) into the server. The comparison between our pipeline and MG-RAST showed that our pipeline provided, after CD-HIT, a much higher proportion of mapped queries (69% versus 3.2%), probably due to the use of the MetaHIT-2014 database, which only contains genes from the human gut microbiome. This result confirms the necessity to use specific databases. In terms of runtime, after sending three time our dataset for analysis, it took two, three and seven days for MG-RAST to send us back the results, which is much longer than our pipeline (around 2–3 hours). Since MG-RAST is a web application, the time needed to obtain the results depends on several parameters. The Internet connection speed of the users will condition the time needed to upload their dataset. Next, the speed of the analysis will depend on the priority assigned to the project, the size of the dataset and the current server load (as specified by the MG-RAST user manual). Furthermore, the MG-RAST does not provide yet any tools for comparing gene expression levels and we believe that our pipeline would be also more convenient for large metatranscriptomic projects in terms of runtime, providing that the users can handle the analysis through the locally installed pipeline.

## Discussion

In this study, the results of the 16S rRNA analysis, which characterizes active bacteria, contrasted with those of 16S rDNA, thereby indicating that not all microorganisms identified at the DNA level play an active role in the gut community. Furthermore, active microbes such as Bifidobacteriaceae showed an increase in relative abundance as an effect of a flatulogenic/high fiber diet, which supports the link between a fiber-enriched diet and saccharolytic bacteria. The functional analysis indicated that a flatulogenic diet significantly up-regulated and down-regulated several metabolic pathways. In order to confirm these results, a greater sample size may be required in future studies. Unexpectedly, in contrast to a strict fiber diet, a flatulogenic diet, which increases the volume of intestinal gas in both subjects complaining of excessive gas production^9^, appeared to decrease several categories of functions that are involved in carbohydrate or energy production. Finally, the observed correlation between volume of gas produced and *Bifidobacterium longum* and several functions and categories of functions could be compared in future studies involving strict plant-based or animal based diet.

For future studies, we recommend to combine DNA-seq with RNA-seq in order to normalize RNA to DNA (i.e. transcripts per gene) when calculating differential expression between samples. Furthermore, in order to recover both 16S rRNA and mRNA sequences in a non-biased manner and to increase the number of potential mRNA reads at a reasonable cost, we recommend using the same total RNA-extracted sample in two separate experiments: 1) a rRNA removal procedure to enrich mRNA sequences and sequencing at a coverage depth of 10–20 million reads per sample; and 2) a sequencing step with a much lower coverage (100,000 reads per sample) without the rRNA removal step to analyze the active microbial composition in an unbiased manner.

Here we designed, implemented, and validated a metatranscriptomic pipeline by making use of the multi-threading capacity of modern computers and then validated its functionality by comparing different methods for taxonomy profiling, by analyzing synthetic mock communities, by analyzing published RNA-seq data and by generating RNA-seq data from fecal samples. The pipeline was implemented on the basis of a constantly changing environment, thus offering the possibility to easily integrate third-party tools, improve parts of the pipeline or change entire modules as long as the input/output folder structure is preserved. The pipeline is available and downloadable from the following webpage: www.metatrans.org, which also provides a tutorial for users.

## Methods

### Ethics Statement

The methods were carried out in accordance with the approved guidelines. All the experimental protocols were approved by the Institutional Review Board of the Vall d’Hebron Hospital (Barcelona, Spain). Subjects provided their written informed consent to participate in this study.

### Synthetic mock communities for validation

To evaluate MetaTrans predictive accuracy for functional analysis, two synthetic mock communities with different expression levels were constituted. Five most abundant microbial genomes were selected based on Qin *et al*.^20^ and were downloaded from the NCBI database: *Bacteroides vulgatus* ATCC 8482, *Ruminococcus torques* L2−14, *Faecalibacterium prausnitzii* SL3/3, *Bacteroides thetaiotaomicron* VPI-5482, *Parabacteroides distasonis* ATCC 8503. A subsample of 1000 genes from each of these microorganisms was selected randomly without replacement to generate a synthetic mock community (4943 reads or 5 Mbp). This mock community was then injected into the Polyester tool^21^ to simulate two groups of samples with differential expression level; with each group containing 50 simulated samples as follow: a different expression level has been simulated in one of the two groups, such that 20% of the genes presented a 4-fold overexpression and 20% a 4-fold underexpression.

To test the accuracy of our pipeline for taxonomic assignment, we used one of the 16S rDNA synthetic mock communities provided by the study of Jeraldo *et al*.^22^ that resembles an ecological sample in terms of composition and abundance. From this original dataset, we used 2500 unique organisms (14800 reads or 21 Mbp) to simulate differential expression with two replicates of 25 samples each, using Polyester. As for the functional simulation, a different expression level was applied in one of the two groups, such that 20% of the genes presented a 4-fold overexpression and 20% a 4-fold underexpression.

Polyester produced then an output of two groups of samples with a different expression level. To simulate reads with quality scores, we used the ART simulator^23^ to produce an equal number of reads in FASTQ format to those produced by Polyester. ART was initially trained with our 8 total RNA samples sequenced in a Hi-Seq 2000 Illumina (see below) to obtain a quality error model. After simulating FASTQ files we then extracted the quality data and bound it to the FASTA files generating new FASTQ files. The R code of the simulations using Polyester was added as a supplementary method (Supplementary Information).

A total of 100 samples for the functional simulation and 50 samples for the taxonomic simulation were then loaded and processed in MetaTrans. To prevent overestimates of accuracy based solely on well-known genomes, we removed from the MetaHIT database those reads that had more than 90% identity with the MetaHIT genes.

To construct ROC curves, we first computed a score (1 – nominal-pvalue) for each gene, which allowed us to rank the genes in order of significance or evidence for differential expression between two groups. The score was two sided, that is, it was not affected by the direction of differential expression between the two conditions. Given a threshold value for such a score, we called all genes with scores exceeding the threshold DE (differentially expressed), and correspondingly all genes with scores below the threshold were called non-DE (non-differentially expressed). Considering the genes that were simulated to be DE as the true positive group and the remaining genes as the true negative group, we computed the false positive rate and the true positive rate for all possible score thresholds and constructed a ROC (Receiver Operating Characteristic) curve for the method.

### Study design and samples collection protocol

In the “total RNA” experiment, stools were collected from four participants—two healthy and two diagnosed with a FBD and complaining of excessive gas evacuation. The subjects were instructed to follow their usual diet for 3 days and to consume a diet rich in fermentable residues for another 3 days, during which each meal (breakfast, lunch, dinner) included at least one portion of the following: (a) bread, cereals or pastries made of whole wheat or corn; (b) beans, soya bean, corn, broad beans, or peas; (c) brussels sprouts, cauliflower, broccoli, cabbage, celery, onion, leek, garlic or artichoke; and (d) banana, fig, peach, grapes or prunes. The volume of intestinal gas was measured as previously described^9^. The gas collection tests were conducted before and after the flatulogenic diet using a rectal balloon catheter (20 F Foley catheter, Bard, Barcelona, Spain) connected via a line without leaks to a barostat, and the volume was continuously recorded (Supplementary Table S5).

In the “rRNA removal” experiment, stools were also collected from four participants – two healthy subjects and two patients with Crohn’s disease.

The stool collection protocol involved providing participants with an ice bag containing an emesis basin (Ref. 104AA200, PRIM S.A, Spain), a 50-mL sterile sampling bottle (Deltalab, Spain), a sterile spatula (Deltalab, Spain), and gloves during their visit to the laboratory. For the purpose of stool collection, participants were instructed to do the following at home: 1) use the emesis basin provided to collect the stool; 2) after the deposit, transfer it to the sampling bottle, ensuring proper homogenization; and 3) take the sampling bottle to the laboratory within the first three hours after deposit or, if not possible, store it in the home freezer (−20 °C) and take it to the laboratory properly surrounded by frozen gel blocks as soon as possible.

Once in the laboratory, the samples were stored at −80 °C until processed as described below.

### DNA extraction and 16S rRNA pyrosequencing from extracted DNA

Sequences of 16S rDNA were recovered from material used in our previous study^9^.

### Total RNA extraction

For total RNA extraction, we used the protocol described in Cardona *et al*.^24^. Briefly, 250 mg of fecal sample was mixed with 500 μl of TE buffer, 0.8 g of Zirconia/silica Beads, 50 μl of SDS 10% solution, 50 μl of sodium acetate, and 500 μl of acid-phenol. Physical disruption was achieved using a FastPrep apparatus (FP120, 101Thermo). Following centrifugation of the lysate, nucleic acids were recovered from the aqueous phase and re-extracted with chloroform:isoamylalcohol. DNA was selectively digested, and RNA was purified using the RNeasy® mini kit (Cat. No. 74104, Qiagen), following the manufacturer’s instructions. Total RNA for an equivalent of 1 mg of each fecal sample was quantified using a Nanodrop ND-1000 Spectrophotometer (Nucliber) while the quality was assessed using a RNA6000 Nano chip (total RNA) in an Agilent 2100 Bioanalyzer. RNA quality was determined by the RNA integrity number (RIN), which is calculated from the relative height and area of the 16S and 23S RNA peaks and follows a numbering system from 1 to 10, 1 being the most degraded profile and 10 the most intact. The average RIN number obtained was 6.8, with values ranging from 6.3 to 7.4.

### rRNA removal and cDNA synthesis and sequencing

For the “rRNA removal” experiment, total RNA of four samples was subjected to an rRNA removal procedure using the Ribo-zero Magnetic kit according to the manufacturer’s instruction (Epicentre, an Illumina® company). After fragmentation of 50 ng of RNA molecules, complementary DNA (cDNA) of the RNA was synthesized using the ScriptSeqTM v2 RNA-Seq Library Preparation Kit from Epicentre with random hexamers, and incorporation of Illumina platform-specific 3′ sequencing tag. The multiplexing index was added through 12 cycles of PCR performed using the FailSafeTM PCR Enzyme Mix (Epicentre Biotechnologies, #FSE51100) followed by AMPure XP Purification (Agencourt, Beckman Coulter). Each library was sequenced as paired-end 76-bp reads on the Illumina HiSeq 2000 platform (National Center for Genome Analysis, Barcelona, Spain) and produced 16 files (8 paired-end), which generated a total of 24 Gbp. NCBI SRA id: PRJNA295252.

### Sequence analysis steps

From the Illumina platform, we obtained paired-end reads in FASTQ format (CASAVA 1.8, Phred +33) separated into distinct files for each single-end read and for each sample. The analysis was performed in four major steps described as such in Fig. 1: filtering, sorting, and functional and taxonomic annotations. The backbone of the pipeline was written in POSIX shell and the internal scripts were written in POSIX, Python, R and AWK languages.

### Filtering of quality reads

The raw reads were submitted to the quality control report-tool FastQC (Andrews, 2010, available at http://www.bioinformatics.babraham.ac.uk/projects/fastqc), which allows evaluation of the quality of the reads and selection of the most appropriate filtering parameters, such as the per base N content, the read length, and the per sequence quality score, for downstream quality control analysis of the reads. The Kraken pipeline^25^ was then used to recover quality reads on the basis of the FastQC report. This set of programs is based on efficient multi-threading and a complete set of tools structured in an independent pipeline. They allow not only common cleaning operations such as removal of low quality reads and filtering of reads with low length, but also Poly-A trimming, N-masked base trimming, collapsing of reads, maintenance of read-pairing along the process, and low-complexity filtering, among other features. This pipeline can be adapted to reads obtained from various sequencing platforms. We set up the configuration of the Kraken tools to maintain the link between paired-end reads during the process and to perform two steps. The first, called “reaper”, relied on the call of a fast and flexible tool designed specifically for short-read processing to trim or remove adapters, as well as to test all reads processed against three criteria: trimming cluster-N regions and removing low quality regions (below a Phred score of 10) and reads with a length <30 nt. The main task of the second step, named “filter”, was to discard reads that had no counterpart and then collapse all identical reads. The header of the read was then modified to include the number of copies of each collapsed read. Finally, the collapsed reads obtained were again subjected to FastQC in order to validate their quality. At this point, if the default quality setting does not cover the quality requirements, the parameters can be refined before analyzing more samples.

### Sorting

After a quality control of reads, to identify those that were clearly non-rRNA/tRNA and therefore potential mRNA, we used an efficient and parallel tool, namely “SortMeRNA”^26^, which required rRNA databases such as SILVA v115^27^, Rfam^28^, and the Genomic tRNA database^29^. Using these three databases, reads were grouped into various categories, namely 16S/18S-rRNA, 23S/28S-rRNA, 5S-rRNA, and tRNA, respectively. As outputs, SortMeRNA produced a file for each category, and the unclassified reads were saved in a separate file as non-rRNA/tRNA, that is to say potential mRNA.

### Functional annotation

We generated paired-end reads for each DNA fragment. Therefore, reads classified as “potential mRNA reads” were first subjected to an overlapping step that merged, when possible, the paired-end reads producing a longer read length. This step was performed using the Fastq-Join tool (Erik Aronesty, 2011: http://code.google.com/p/ea-utils) with a minimum overlap of 8 bp and a maximum difference of 10%, as proposed in the MG-RAST pipeline (18). The potential mRNA reads file may still contain a number of undesired sequences that do not provide functional information, such as non-coding regions, and should therefore be removed in order to decrease computation time in downstream analysis. For this step, we used FragGeneScan (v.1.17)^30^ to predict putative genes and discard the rest. This tool was configured with appropriate parameters to work properly with relatively short reads such as those of Illumina. Predicted genes were then subjected to clustering to further reduce the size of the dataset using CD-HIT v4.6 with an identity threshold of >= 95% and a gene overlap of >= 90%. Information on cluster size was then included in the header of all representative reads. Finally, to recover a functional profile for each sample, the potential mRNA reads were mapped against a functional database such as the latest MetaHIT gene catalog^2^ using SOAP2^10^, with the first match retained. The MetaHIT-2014 database contains functions that were recovered from about 1250 human gut microbiome samples and that were annotated with the EggNOGv3 functional database. In order to use a more general database to analyze for instance other ecosystems than the gut microbiota, we added to the pipeline another database, M5nr and the possibility to use either M5nr or the MetaHIT-2014 database. The M5nr database^12^ is a non-redundant protein database provided by the MG-RAST server and contains 15.9 million unique proteins and 5.8 million functional annotations from different sources including Integrated Microbial Genomes (IMG), Genbank, InterPro, Kyoto Encyclopedia of Genes and Genomes (KEGG), PathoSystems Ressource Integration Center (PATRIC), Phage Annotation Tools and Methods (Phantome), Reference Sequence (RefSeq), the SEED Project, UniProt Knowledgebase (UniProt). An in-house script was used to take into account cluster sizes and to discard duplicates. Raw abundance matrices were generated and processed using the DESeq2 package^31^ to uncover the most differentially expressed functions. Up- or down-regulated functions were further plotted into metabolic pathways using iPath2^32^.

### Taxonomic annotation

In the “total RNA” experiment from the paired-end read files previously classified as rRNA/tRNA, the two single reads from the DNA fragment were overlapped using Fastq-Join to increase read lengths and annotation accuracy. From the file containing all overlapped reads for each sample, we randomly extracted 100,000 using a reservoir sampling method without replacement to reduce computational time. Next, these sequences were clustered using the UCLUST method^17^ and mapped by homology using SOAP2 against the 16S rRNA Greengenes v13.5 database^11^ and only best hits were retained for further analysis. An abundance raw-count table was built for the seven taxonomical ranks, from phylum to species levels for all samples. In the tables we removed all singleton elements (those appearing just once in a sample) to avoid false positive assignments and then sorted all elements in descending order on the basis of their abundance average using awk and shell scripts.

### Comparison of microbial diversity between 16S rRNA and 16S rDNA

In parallel, we analyzed 16S rDNA amplicon files obtained in a previous study^9^ by pyrosequencing the same fecal samples using the Quantitative Insights Into Microbial Ecology pipeline (QIIME v1.8)^33^. The 16S rDNA sequences were quality-filtered by applying default settings and a minimum acceptable average Phred score of 25 in windows of 50 continuous nucleotides. Using the pick-otus script, we classified the sequence reads into OTUs (Operational Taxonomic Units) on the basis of sequence similarity using the UCLUST algorithm^17^. PyNAST was used to align each OTU against a core set of sequences representative of most of the major prokaryotic taxa from the Greengenes v13.5 database)^34^. We then identified and removed chimeric sequences using the ChimeraSlayer algorithm^35^. Remaining clean OTUs were taxonomically annotated against the Greengenes v13.5 database using BLASTN. The script make_phylogeny.py was used to create phylogenetic trees using the FastTree^36^ method. To correctly define species richness for the analysis of between-sample diversity, known as beta diversity, the OTU table was rarefied at 1,952 sequences per sample. Rarefaction is used to overcome cases in which read counts are not similar in numbers between samples. In order to avoid false positive taxa, we considered only taxa that presented at least 0.2% of the sequences in at least one sample. The summarized taxa feature was used to classify taxa from the Domain to the Species level. To calculate between-sample diversity, weighted and unweighted UniFrac metrics were applied to build phylogenetic distance matrices, which were then used to construct hierarchical cluster trees using PCoA representations (Principal Coordinate Analysis). To make abundance tables comparable between the two experiments, we also rarefied abundance tables from rRNA datasets at 1,952 sequences per sample and also removed any taxa annotation that did not contain at least 0.2% of the signal. Statistical analyses were carried out in R using the Friedman test, a non-parametric paired-test used to compare the mean number of sequences of the groups of experiments (16S rDNA vs. 16S rRNA and before and after diet challenge). Since we used non-parametric correlations, significance was determined through permutations. The analysis provided false discovery rate (FDR)-corrected P-values (q).

## Additional Information

**Accession code**: The sequences will be uploaded to NCBI-SRA at publication.

**How to cite this article**: Martinez, X. *et al*. MetaTrans: an open-source pipeline for metatranscriptomics. *Sci. Rep.* **6**, 26447; doi: 10.1038/srep26447 (2016).

## Supplementary Material

Supplementary Information
