# Supplementary material for: MetaTrans: an open-source pipeline for metatranscriptomics
Source: Sci Rep. 2016 May 23;6:26447. doi: 10.1038/srep26447 (PMC4876386; doi:10.1038/srep26447)
Supplement: Supplementary Information [file srep26447-s1.doc]

**SUPPLEMENTARY INFORMATION**

**MetaTrans: an open-source pipeline for metatranscriptomics**

Xavier Martinez^1^, Victoria Pascal^1^, Marta Pozuelo^1^, David Campos^1^, Ivo Gut^2,3^, Marta Gut^2,3^, Fernando Azpiroz^1,4^, Francisco Guarner^1,4^, Chaysavanh Manichanh^1,4,*^

^1^Digestive Research Unit, Vall d’Hebron Research Institute, Barcelona, 08035, Spain

^2^CNAG-CRG, Centre for Genomic Regulation (CRG), Barcelona, 08028, Spain

^3^Universitat Pompeu Fabra (UPF), 08002, Barcelona, Spain

^4^CIBERehd, Instituto de Salud Carlos III, Madrid, 28029, Spain

* To whom correspondence should be addressed. Tel: +34934894036; Fax: +34934894032; Email: cmanicha@gmail.com.

**Supplementary Information**

###Simulate RNA-seq experiment using negative binomial model###

#########################################################################

# Metatrans - metatranscriptomic sequence analysis pipeline for the human gut microbiota. #

# #

# Copyright (C) 2015 Xavier Martinez VHIR - Vall d'Hebron #

# Institut de Recerca #

# #

# This file is part of Metatrans. #

# #

# Metatrans is free software: you can redistribute it and/or modify #

# it under the terms of the GNU General Public License as published by #

# the Free Software Foundation, either version 3 of the License, or #

# (at your option) any later version. #

# #

# Metatrans is distributed in the hope that it will be useful, #

# but WITHOUT ANY WARRANTY; without even the implied warranty of #

# MERCHANTABILITY or FITNESS FOR A PARTICULAR PURPOSE. See the #

# GNU General Public License for more details. #

# #

# You should have received a copy of the GNU General Public License #

# along with Metatrans. If not, see <http://www.gnu.org/licenses/>. #

# #

# Contact e-mail: #

# metatrans-forum@googlegroups.com #

#########################################################################

############### PACAKGE INFO ###############

#(Vignette: file:///home/adminuser/R/x86_64-pc-linux-gnulibrary/

3.1/polyester/doc/polyester.html :

#vignette("polyester")

# For both simulate_experiment and simulate_experiment_countmat, you can change

these parameters:

# fraglen: Mean fragment length (default 250)

# fragsd: Standard devation of fragment lengths (default 25)

# readlen: Read length (default 100)

# error_rate: Sequencing error rate: probability that the sequencer records the

wrong

nucleotide at any given base (default 0.005, uniform error model assumed)

#paired: Whether the reads should be paired-end (default TRUE)

#R version: 3.2.3

#Polyester version: 1.6.0

############################################

#source("http://bioconductor.org/biocLite.R")

#biocLite("polyester")

library(polyester)

library(Biostrings)

path <- "/home_path/source_fasta_file"

fasta_file = paste0(path,"/5_organisms_1000_genes.fasta")

ptm <- proc.time() #Set clock to get the runtime

default_wd<-getwd()

numtx=14803 #Number of reads in the source fasta file

path <- "/home_path/polyester_simulation"

setwd(path)

outdir = paste0('simulation-variation20-50techreplicates-',numtx)

#E.g. Simulation at ~20x coverage:

#reads per transcript = length/readlength * 20

#length=width(small_fasta) -> length of each transcripts

#readlength=100

#coverage=20 -->"simulate from the first 20 of these transcripts"

# "width" is operating on a DNAStringSet (from Biostrings)

#readspertx = round(coverage * width(fasta) / readlen)

#reads_per_transcript: The baseline mean number of reads for each transcript.

### Coverage 20% setup (with 2 groups of 3 samples each)

#HELP:

http://bioconductor.org/packages/release/bioc/manuals/polyester/man/polyester.pdf

#Experiment design:

nreps=c(50,50) #2 groups of 50 replicates each.

error_rate=0.005 #5% uniform error probability (default)

paired=TRUE #paired-end samples

coverage=5

fasta = readDNAStringSet(fasta_file)

readlen=76 #reads of 76bp

readspertx=round(coverage * width(fasta) / readlen)

#Variation at 20% up/down genes:

#FoldChange=4

#Down: fc=b/a=1/4 -> b=a/4 -> *0.25

#Up: fc=b/a=4 -> b=4*a -> *4

#Variation 20:

# 20%down, 60% , 20%up

set.seed(12)

fold_changes=cbind( rep(1,numtx),

sample( c(0.25, 1, 4),

size=numtx,

prob=c(0.2, 0.6, 0.2),

replace=TRUE)

)

#Set seed for reproducibility

seed=142

simulate_experiment(fasta_file,

reads_per_transcript=readspertx,

num_reps=nreps,

fold_changes=fold_changes,

outdir=outdir,

paired=paired,

readlen=readlen,

error_rate=error_rate,

seed=seed)

proc.time() - ptm #Print time used by the process

**Supplementary Figures**

**Figure S1. Pipeline validation.** In order to test whether our pipeline provided similar results to those obtained using a previously reported tool, we analyzed part of a published dataset (Leimena et al., 2013) using our pipeline. We obtained similar functional categories (left) to those described in Leimena *et al.* 2013 (right).

**
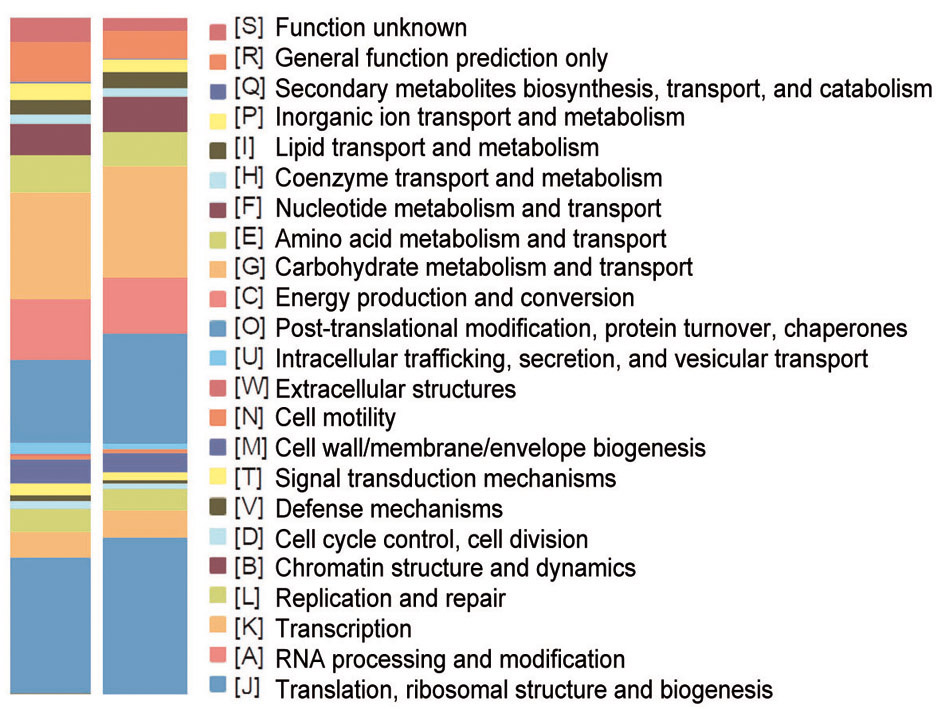
**

**Figure S2. Similarity in functional mapping between BLASTP, DIAMOND-BLASTP** and SOAP2 against the MetaHIT-2014 database using dataset from sample #1_BF as shown by a Venn diagram (**a**) and the plot of the total number of unique IDs that have a match against the MetaHIT-2014 database (**b**).

**
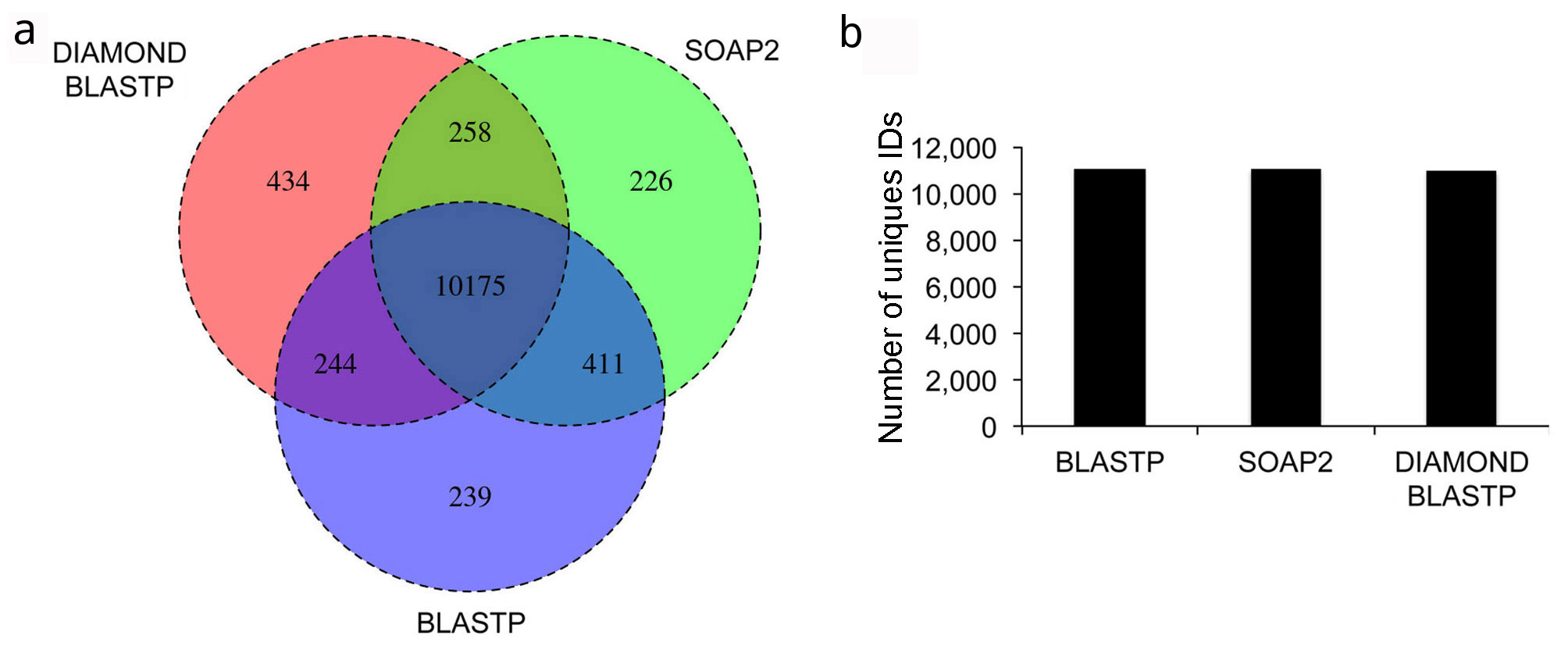
**

**Figure S3. Taxonomy profiling of the 16S rRNA library at the phylum, family and genus levels.** Only groups of microbes accounting for more than 0.01% and 0.1% of the total sequences at family and genus level, respectively, were represented in the graphs.


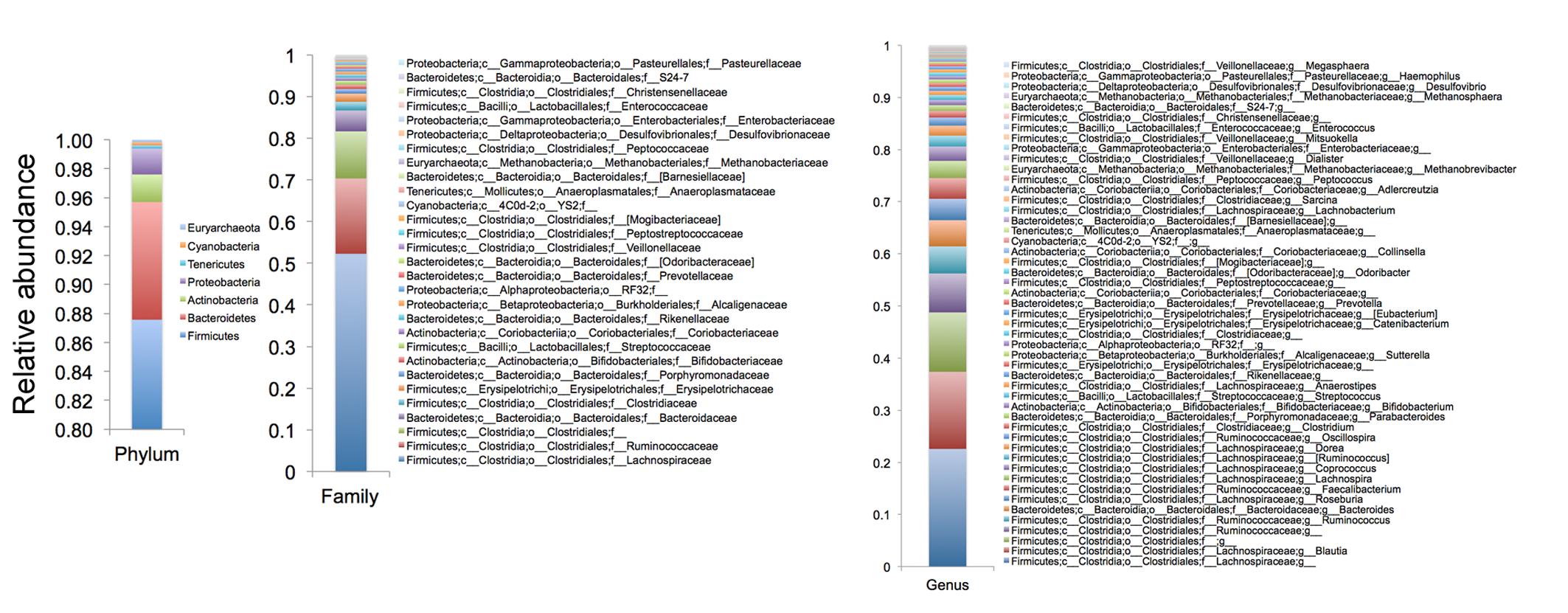


**Supplementary Tables**

**Table S1. Description of the outputs from each analysis step of the “total RNA” experiment.**

|  | **#1_BF Diet** | **#1_AF Diet** | **#2_BF Diet** | **#2_AF Diet** | **#3_BF Diet** | **#3_AF Diet** | **#4_BF Diet** | **#4_AF Diet** | **Average** |
| --- | --- | --- | --- | --- | --- | --- | --- | --- | --- |
| **Raw reads (X2)** | 39534526 | 44137900 | 36621522 | 35376906 | 44550458 | 46221230 | 29230252 | 43061504 | 39841788 |
| **After quality control (%)** | 80 | 78 | 79 | 75 | 77 | 76 | 78 | 82 | 78.1 |
| **rRNA/tRNA (%)** | 75 | 73 | 76 | 72 | 76 | 71 | 73 | 77 | 74.1 |
| **Non rRNA/tRNA (%)** | 5.7 | 5.2 | 3.0 | 3.1 | 1.4 | 5.0 | 5.8 | 5.3 | 4.3 |
| **After paired-end overlapping (%)** | 4.7 | 4.2 | 2.4 | 2.5 | 1.2 | 4.4 | 4.7 | 4.5 | 3.6 |
| **After FragGeneScan (%)** | 3.9 | 3.4 | 1.8 | 2.0 | 1.0 | 3.5 | 4.0 | 3.8 | 2.9 |
| **After CD-HIT (%)** | 2.7 | 2.3 | 1.0 | 1.3 | 0.6 | 2.6 | 2.8 | 2.4 | 2.0 |
| **MetaHIt ids** | 965435 | 906270 | 350852 | 438399 | 235689 | 1072589 | 805893 | 1077089 | 731527 |
| **Unique MetaHit ids** | 292371 | 288398 | 111463 | 140293 | 85343 | 278887 | 201651 | 252232 | 206330 |

**Table S2.** **Description of the outputs from each analysis step of the “rRNA removal” experiment.**

|  | **#5** | **#6** | **#7** | **#8** | **Average** |
| --- | --- | --- | --- | --- | --- |
| **Raw reads (X2)** | 50948448 | 51008628 | 61034072 | 55883372 | 54718630 |
| **After quality control (%)** | 40.6 | 67.1 | 62.3 | 49.6 | 54.9 |
| **rRNA/tRNA (%)** | 3.7 | 4.8 | 0.7 | 1.4 | 2.7 |
| **Non rRNA/tRNA (%)** | 36.9 | 62.3 | 61.7 | 48.3 | 52.3 |
| **After paired-end overlapping (%)** | 26.8 | 45 | 44.3 | 35.5 | 37.9 |
| **After FragGeneScan (%)** | 23.8 | 40.9 | 41.6 | 32.5 | 34.7 |
| **After CD-HIT (%)** | 4 | 7.2 | 6.7 | 4.6 | 5.6 |
| **MetaHIt ids** | 7010023 | 12337711 | 16396625 | 10614486 | 11589711 |
| **Unique MetaHit ids** | 221132 | 482156 | 495872 | 320431 | 379898 |

**Table S3. Number of orthologous IDs for each COG functional categories, after mapping the putative genes against the MetaHIT-2014 database.**

| COG functional categories | Average number of orthologous IDs |
| --- | --- |
| unknown_funcat_id unknown | 149910 |
| [S] Function unknown | 77302 |
| [G] Carbohydrate transport and metabolism | 58027 |
| [R] General function prediction only | 47641 |
| [L] Replication, recombination and repair | 41075 |
| [M] Cell wall/membrane/envelope biogenesis | 30818 |
| [E] Amino acid transport and metabolism | 30396 |
| [K] Transcription | 30403 |
| [J] Translation, ribosomal structure and biogenesis | 29237 |
| [T] Signal transduction mechanisms | 26121 |
| [C] Energy production and conversion | 26640 |
| [V] Defense mechanisms | 15434 |
| [P] Inorganic ion transport and metabolism | 14965 |
| [O] Posttranslational modification, protein turnover, chaperones | 14452 |
| [H] Coenzyme transport and metabolism | 11814 |
| [F] Nucleotide transport and metabolism | 10741 |
| [I] Lipid transport and metabolism | 8656 |
| [U] Intracellular trafficking, secretion, and vesicular transport | 8212 |
| [N] Cell motility | 6162 |
| [D] Cell cycle control, cell division, chromosome partitioning | 5955 |
| [Q] Secondary metabolites biosynthesis, transport and catabolism | 3949 |
| [Z] Cytoskeleton | 363 |
| [B] Chromatin structure and dynamics | 27 |
| [W] Extracellular structures | 22 |
| [A] RNA processing and modification | 10 |
| [Y] Nuclear structure | 0 |

**Table S4.** **Correlation between volume of intestinal gas and microbiome functions.**

| **Positive Correlation** |  |  |  |
| --- | --- | --- | --- |
| **Protein_identifier** | Positive_correlation (r) | p-value | eggNOG_function |
| **COG1124** | 0.873 | 0.005 | ABC-type dipeptide/oligopeptide/nickel transport system, ATPase component |
| **COG1108** | 0.917 | 0.001 | ABC-type Mn2+/Zn2+ transport systems, permease components |
| **NOG149457** | 0.866 | 0.005 | Acetylornithine deacetylase |
| **COG2087** | 0.928 | 0.001 | Adenosyl cobinamide kinase/adenosyl cobinamide phosphate guanylyltransferase [..] |
| **COG0483** | 0.845 | 0.008 | Archaeal fructose-1,6-bisphosphatase and related enzymes of inositol monophosphatase [..] |
| **COG4385** | 0.909 | 0.002 | Bacteriophage P2-related tail formation protein |
| **COG0502** | 0.857 | 0.007 | Biotin synthase and related enzymes |
| **bactNOG07342** | 0.846 | 0.008 | Biotin-(Acetyl-Coa carboxylase) ligase |
| **COG2272** | 0.873 | 0.005 | Carboxylesterase type B |
| **bactNOG62474** | 0.839 | 0.009 | Diguanylate cyclase with PAS/PAC sensor |
| **NOG147270** | 0.896 | 0.003 | Endonuclease |
| **NOG69508** | 0.873 | 0.005 | Flavocytochrome c heme subunit |
| **COG1760** | 0.892 | 0.003 | L-serine deaminase |
| **COG3407** | 0.854 | 0.007 | Mevalonate pyrophosphate decarboxylase |
| **NOG253312** | 0.881 | 0.007 | Microtubule binding protein |
| **COG1149** | 0.846 | 0.008 | MinD superfamily P-loop ATPase containing an inserted ferredoxin domain |
| **NOG115567** | 0.857 | 0.007 | Mobilization protein |
| **firmNOG04823** | 0.913 | 0.002 | N-Acetylmuramoyl-L-Alanine amidase |
| **NOG68179** | 0.862 | 0.006 | Peptidase C14 caspase catalytic subunit P20 |
| **bactNOG13740** | 0.836 | 0.010 | Peptidase S9, prolyl oligopeptidase active site domain protein |
| **COG3503** | 0.917 | 0.001 | Predicted membrane protein |
| **COG1971** | 0.858 | 0.006 | Predicted membrane protein |
| **COG1808** | 0.849 | 0.008 | Predicted membrane protein |
| **COG3164** | 0.846 | 0.008 | Predicted membrane protein |
| **COG1058** | 0.910 | 0.002 | Predicted nucleotide-utilizing enzyme related to molybdopterin-biosynthesis enzyme [..] |
| **COG1827** | 0.865 | 0.006 | Predicted small molecule binding protein (contains 3H domain) |
| **NOG42679** | 0.849 | 0.008 | Protein found in conjugate transposon |
| **NOG44942** | 0.893 | 0.003 | Protein found in conjugate transposon TraI |
| **NOG04815** | 0.888 | 0.003 | Protein involved in cell division |
| **COG1457** | 0.962 | 0.000 | Purine-cytosine permease and related proteins |
| **COG2946** | 0.857 | 0.011 | Putative phage replication protein RstA |
| **bactNOG12111** | 0.838 | 0.009 | Recombinase |
| **bactNOG89275** | 0.858 | 0.006 | Relaxase/Mobilization nuclease |
| **NOG150680** | 0.857 | 0.011 | Relaxase/Mobilization nuclease family protein |
| **NOG42738** | 0.873 | 0.005 | Replication protein |
| **bactNOG61245** | 0.880 | 0.004 | Reverse transcriptase |
| **COG2897** | 0.910 | 0.002 | Rhodanese-related sulfurtransferase |
| **COG0741** | 0.860 | 0.006 | Soluble lytic murein transglycosylase and related regulatory proteins (some contain [..] |
| **COG2969** | 0.837 | 0.010 | Stringent starvation protein B |
| **NOG131827** | 0.927 | 0.001 | Sulfurtransferase |
| **NOG245485** | 0.846 | 0.008 | Synaptosomal-Associated protein |
| **NOG44493** | 0.852 | 0.007 | Terminase large subunit |
| **NOG128913** | 0.938 | 0.001 | Terminase, large subunit |
| **NOG45696** | 0.879 | 0.004 | TMEM229B (human): transmembrane protein 229B |
| **NOG117686** | 0.877 | 0.004 | Tmem229b (mouse): transmembrane protein 229B Gene |
| **COG3505** | 0.846 | 0.008 | Type IV secretory pathway, VirD4 components |
| **COG0829** | 0.923 | 0.001 | Urease accessory protein UreH |
| **COG3309** | 0.870 | 0.005 | Uncharacterized virulence-associated protein D |
| **COG2022** | 0.869 | 0.005 | Uncharacterized enzyme of thiazole biosynthesis |
| **COG1633** | 0.869 | 0.005 | Uncharacterized conserved protein |
| **COG1268** | 0.858 | 0.006 | Uncharacterized conserved protein |
| **COG2135** | 0.848 | 0.008 | Uncharacterized conserved protein |
| **COG3797** | 0.867 | 0.005 | Uncharacterized protein conserved in bacteria |
| **COG2911** | 0.842 | 0.009 | Uncharacterized protein conserved in bacteria |
| **COG3544** | 0.837 | 0.010 | Uncharacterized protein conserved in bacteria |
| **NOG288370** | 0.959 | 0.000 | unknown |
| **NOG116027** | 0.987 | 0.000 | unknown |
| **firmNOG09351** | 0.946 | 0.000 | unknown |
| **NOG42974** | 0.940 | 0.001 | unknown |
| **NOG261952** | 0.934 | 0.001 | unknown |
| **NOG114522** | 0.929 | 0.001 | unknown |
| **NOG29070** | 0.917 | 0.001 | unknown |
| **NOG05556** | 0.917 | 0.001 | unknown |
| **NOG129911** | 0.914 | 0.001 | unknown |
| **NOG10085** | 0.895 | 0.003 | unknown |
| **NOG303381** | 0.892 | 0.003 | unknown |
| **NOG286363** | 0.884 | 0.004 | unknown |
| **NOG262245** | 0.879 | 0.004 | unknown |
| **NOG45601** | 0.873 | 0.005 | unknown |
| **NOG258560** | 0.871 | 0.005 | unknown |
| **NOG80481** | 0.855 | 0.007 | unknown |
| **NOG39171** | 0.855 | 0.007 | unknown |
| **NOG280103** | 0.855 | 0.007 | unknown |
| **bactNOG94537** | 0.853 | 0.007 | unknown |
| **NOG126111** | 0.841 | 0.009 | unknown |
| **NOG81594** | 0.839 | 0.009 | unknown |
| **NOG145253** | 0.838 | 0.009 | unknown |
| **NOG11519** | 0.913 | 0.002 | unknown |
| **NOG09683** | 0.888 | 0.003 | unknown |
| **NOG250051** | 0.888 | 0.003 | unknown |
| **NOG114383** | 0.873 | 0.005 | unknown |
| **NOG263917** | 0.857 | 0.011 | unknown |
| **NOG239904** | 0.854 | 0.007 | unknown |
| **NOG288558** | 0.846 | 0.008 | unknown |
| **firmNOG13705** | 0.846 | 0.008 | unknown |
| **NOG145890** | 0.846 | 0.008 | unknown |
| **bactNOG43603** | 0.846 | 0.008 | unknown |
| **NOG300337** | 0.846 | 0.008 | unknown |
| **NOG260360** | 0.846 | 0.008 | unknown |

| **Negative Correlation** |  |  |  |
| --- | --- | --- | --- |
| **Protein_identifier** | Negative_correlation (r) | p-value | eggNOG_function |
| **COG1871** | -0.863 | 0.006 | Chemotaxis protein; stimulates methylation of MCP proteins |
| **NOG137762** | -0.836 | 0.010 | Cobalt ABC transporter, inner membrane subunit CbiQ |
| **COG0202** | -0.881 | 0.007 | DNA-directed RNA polymerase, alpha subunit/40 kD subunit |
| **NOG09687** | -0.905 | 0.005 | Flagellar operon protein |
| **COG0014** | -0.885 | 0.003 | Gamma-glutamyl phosphate reductase |
| **NOG247644** | -0.841 | 0.009 | Protein, partial |
| **COG0360** | -0.905 | 0.005 | Ribosomal protein S6 |
| **COG1799** | -0.904 | 0.002 | Uncharacterized protein conserved in bacteria |
| **NOG131207** | -0.845 | 0.008 | unknown |
| **NOG130001** | -0.851 | 0.007 | unknown |
| **NOG249219** | -0.871 | 0.005 | unknown |
| **NOG40611** | -0.893 | 0.003 | unknown |
| **NOG296983** | -0.952 | 0.000 | unknown |
| **NOG139651** | -0.862 | 0.006 | unknown |

**Table S5. Volume of intestinal gas recorded using a rectal balloon catheter connected via a line without leaks to a barostat.**

|  | **Before Diet (ml)** | **After Diet (ml)** |
| --- | --- | --- |
| **#1** | 284 | 446 |
| **#2** | 410 | 1621 |
| **#3** | 167 | 967 |
| **#4** | 135 | 573 |
